# Integrating Teaching and Research in Undergraduate Biology Laboratory Education

**DOI:** 10.1371/journal.pbio.1001174

**Published:** 2011-11-15

**Authors:** Matthew J. Kloser, Sara E. Brownell, Nona R. Chiariello, Tadashi Fukami

**Affiliations:** 1 School of Education, Stanford University, Stanford, California, United States of America; 2 Department of Biology, Stanford University, Stanford, California, United States of America; University of California Berkeley/Joint Genome Institute, United States of America

## Abstract

A course recently designed and implemented at Stanford University applies practical suggestions for creating research-based undergraduate courses that benefit both teaching and research.

The dilemma is well known. Scientists at research-focused universities must precariously balance a research agenda while also contributing to the education of undergraduate students [1]–[3]. An imbalance exists at many universities where more time, resources, and prestige are devoted to research at the expense of teaching future generations of scientists and scientifically literate citizens [1],[2]. Indeed, the term “teaching load” suggests that teaching is a burden that diverts time and energy away from productive scholarship. However, this view inaccurately presents teaching and research as a zero-sum game when, in reality, well-designed curricula can benefit both activities [4]–[6]. In this article, we provide practical suggestions for implementing such curricula and describe a recently designed course as an example of how they can be applied.

Both the National Academies and the American Association for the Advancement of Science recently emphasized that undergraduate education could be improved by a higher level of student participation in authentic research [7],[8]. Their recommendations are of two types: early student engagement in research labs, and enrollment in “research-based” (also known as “discovery-based,” “project-based,” and “inquiry-based”) courses modeled on real-world scientific practice. Although both types provide students with an authentic representation of science, research-based courses ensure more structured support and a more consistent lab experience for all students.

The number of research-based lab courses has increased over the past two decades [9],[10], but the traditional “cookbook” labs, in which students follow a given list of procedures, are still prominent features of undergraduate curricula in many institutions [11]. Although changes are beginning to be made, logistical challenges coupled with little motivation for faculty to dedicate much time to teaching remain significant barriers to widespread implementation of research-based courses at the university level [3]. The challenges of implementing these courses are especially acute for high-enrollment classes required for biology majors and pre-med students. Given these challenges, building courses on existing faculty research programs may provide a viable solution at many institutions [12],[13].

Toward this goal, a new undergraduate introductory lab course was recently created by the Department of Biology at Stanford University. An instructional team, led by a tenure-track professor (the fourth author of this article), designed and taught a 10-week lab course that engaged a large student population in authentic research experiences based on one of his current research projects. With a focus on ecology, the course incorporated key components of authentic research, including collaboration among students, utilization of modern research techniques to study longitudinal, open-ended research questions with unknown answers, and scientific communication of results. Student-collected data were in turn incorporated in the instructor's research program.

Using our experience with this course and drawing on the experience of other initiatives outlined in previously published curricula [6],[7],[12],[13], we present six recommendations that could be applied to various biological subdisciplines to develop courses with the dual function of providing students with a research-based experience and contributing to the instructor's research platform (Box 1). These recommendations include: (1) a low barrier of technical expertise needed for students to collect data; (2) established checks and balances to ensure that student mistakes will not compromise research quality; (3) a diverse set of variables that present many combinatorial choices for students to investigate without overwhelming the instructional team; (4) a central standardized database into which students can upload data; (5) assessment measures that are representative of real-world science; and (6) involvement of instructors with expertise in the study system. For others interested in designing this type of course, unique institutional contexts and logistics will likely influence the creation of different courses, but it is our hope that these guidelines and the course we briefly describe below can be used as a template for developing high-enrollment courses based on a faculty research program.

**Box 1. Suggestions for Creating a Research-Based Course Using a Faculty Research Program**

**1. Low barrier of technical expertise for students to collect data**

- Data collection should require minimal prior knowledge or technical skill.
- Technically difficult procedures that cannot be mastered by students quickly can be executed by staff members, but demonstrated to students so they understand the processes behind the data collection.

**2. Established checks and balances for student-collected data**

- Student-collected data should require either minimal expertise or be repeated by a second lab group as a check for data collection accuracy.

**3. Diverse, but constrained set of variables for developing hypotheses**

- The given model system should have enough variables to allow for a variety of student questions.
- The number of variables available to students should be constrained in order to limit the work of the instructional team and increase the common ground on which peer discussions can occur.

**4. Central database accessible to all students**

- A central database allows students to access data from previous years and other lab groups.
- The ever-increasing size of the database provides students with realistic sample sizes that could not be obtained if students only used data generated during the course.

**5. Course assessments reflect authentic scientific communication**

- The final paper should follow the format of an influential journal in the given field, and students should receive multiple iterations of feedback from peers and instructors.
- Students should present their findings in a conference-like presentation format at the end of the course.

**6. Research-specific expertise of faculty member**

- The instructors should leverage their expertise with both general biological concepts and the specific research system in order to foster high-level discussions and provide effective feedback to students.

## Incorporating Research into Curriculum: An Example

The course used a system of four biotic and three abiotic variables surrounding the microorganisms that colonize the floral nectar of the sticky monkeyflower (*Mimulus aurantiacus*) under natural field conditions. Data collection required minimal technical training, allowing students to immediately begin collecting data at a site near the university campus (Jasper Ridge Biological Preserve, Stanford, California). Despite the simple techniques used, students were able to generate and test a variety of hypotheses on ecological interactions (see video at http://www.bio-link.org/home/summer-fellows-forum-2011/mimulus). Students were assigned a set of eight plants for which they collected weekly data that were uploaded to a centralized database. This division of labor increased collaboration among students, provided them with a dataset that could not be collected individually in a 10-week class, and contributed to the ever-expanding dataset that could be used for both research purposes and future iterations of the course.

The centralized dataset allowed students to test unique research questions on the same ecological system. Some of these questions involved the density of yeast present in floral nectar as a function of local temperature, water availability or the number of pollinator visits, and the effect of yeast density on pollination. The results were communicated using authentic scientific modes of dissemination, specifically a journal article-style final paper and a 7-minute conference-like presentation with an equal amount of time allotted for questions. Students had worked on the same general research topic during the course and were thus engaged during the presentations, as evidenced by their high-level conceptual and technical questions. Many students used their own findings to interpret other groups', which resulted in discussion similar to an actual scientific conference. An external evaluation [14] indicated that this research-based course positively affected students' attitudes toward research, self-confidence in performing lab-related tasks, and interest in pursuing future research opportunities. Collaboration was high among students in the lab including frequent conceptual discussions with peers and instructors.

Designing the course on the basis of the instructor's own research program provided two advantages. First, the instructor's expertise helped students form and test interesting hypotheses of scientific merit that go beyond an educational exercise. Second, the course resulted in a source of novel data and hypotheses that are being used by the instructor's research lab to both guide and answer research questions [15],[16].

This course is only one example, focused on just one subdiscipline of biology. However, we believe the recommendations we discussed (Box 1) are general enough to be broadly applicable to various subdisciplines. Data collection for the ecology course involved technically simple skills such as monitoring flowering phenology and downloading temperature data from small probes. Other lab courses may require more complex molecular or cellular techniques. This raises different challenges, but if data are intended to be incorporated in the instructor's research project, it would still be possible if multiple lab groups compared results from the same assays to ensure that the data are accurate. In certain cases, the end results could be verified through other means (i.e., final products could be sequenced to check if cloning was done properly). Creating these types of courses may not be easy and may initially require a large amount of resources, both financial and personnel. However, the benefits to both students and instructors may well make the investments worthwhile.
